# Inter- and Intra-Host Nucleotide Variations in Hepatitis A Virus in Culture and Clinical Samples Detected by Next-Generation Sequencing

**DOI:** 10.3390/v10110619

**Published:** 2018-11-09

**Authors:** Zhihui Yang, Mark Mammel, Chris A. Whitehouse, Diana Ngo, Michael Kulka

**Affiliations:** 1 Office of Applied Research and Safety Assessment, Center for Food Safety and Applied Nutrition, U.S. Food and Drug Administration, Laurel, MD 20708, USA; mark.mammel@fda.hhs.gov (M.M.); diana.ngo@fda.hhs.gov (D.N.); michael.kulka@fda.hhs.gov (M.K.); 2 Office of Research, Center for Veterinary Medicine, U.S. Food and Drug Administration, Laurel, MD 20708, USA; chris.whitehouse@fda.hhs.gov

**Keywords:** inter- and intra-host nucleotide variations, Hepatitis A virus, next-generation sequencing, pyrosequencing

## Abstract

The accurate virus detection, strain discrimination, and source attribution of contaminated food items remains a persistent challenge because of the high mutation rates anticipated to occur in foodborne RNA viruses, such as hepatitis A virus (HAV). This has led to predictions of the existence of more than one sequence variant between the hosts (inter-host) or within an individual host (intra-host). However, there have been no reports of intra-host variants from an infected single individual, and little is known about the accuracy of the single nucleotide variations (SNVs) calling with various methods. In this study, the presence and identity of viral SNVs, either between HAV clinical specimens or among a series of samples derived from HAV clone1-infected FRhK4 cells, were determined following analyses of nucleotide sequences generated using next-generation sequencing (NGS) and pyrosequencing methods. The results demonstrate the co-existence of inter- and intra-host variants both in the clinical specimens and the cultured samples. The discovery and confirmation of multi-viral RNAs in an infected individual is dependent on the strain discrimination at the SNV level, and critical for successful outbreak traceback and source attribution investigations. The detection of SNVs in a time series of HAV infected FRhK4 cells improved our understanding on the mutation dynamics determined probably by different selective pressures. Additionally, it demonstrated that NGS could potentially provide a valuable investigative approach toward SNV detection and identification for other RNA viruses.

## 1. Introduction

The majority of the known foodborne viruses, either linked or found directly responsible for foodborne illness, have RNA genomes [1]. RNA viruses have been shown to exhibit high mutation rates primarily due to the low-fidelity of RNA polymerases [2] and absence of post-replication nucleotide repair mechanisms [3,4]. Therefore, these RNA viruses are generally expected to exist as populations of non-identical but closely genetic-related viral variants between the hosts (inter-host) or within an individual host (intra-host), which are referred to as quasispecies [5,6]. Viral genetic heterogeneity, generated by single nucleotide variations (SNVs), is believed to be a strategy of virus evolution and virus adaptability [4]. However, the presence of more than one sequence variant is a challenge for accurate virus detection, identification, and source attribution of contaminated food items.

Hepatitis A virus (HAV) is one of the identified major foodborne viruses in the U.S. [1,7]. Although the incidence of HAV has declined due to the introduction of HAV vaccines in the 1990s [8,9], the number of cases appears to be increasing in the U.S. (estimated number of new HAV infection in 2015 was 2800, according to the CDC (Center for Disease Control and Prevention): Viral Hepatitis Surveillance United States, 2015), as well as around the world (estimated number of HAV clinical cases was 1.5 million, according to the World Health Organization, 2000). HAV is commonly transmitted person-to-person, or through the consumption of contaminated food or water, and large outbreaks of HAV associated with contaminated food continue to be reported worldwide [10,11,12,13].

HAV is a non-enveloped, RNA virus belonging to the family Picornaviridae, whose single-stranded genome of approximately 7.5 kilobases (kb) in length, contains a single open reading frame encoding a single polyprotein flanked by 5′ and 3′ untranslated regions, as well as a 3′ poly(A) tail [14,15]. Since its discovery in 1973 by Steven Feinstone [16], HAV has evolved through nucleotide mutations and recombination and is classified into three genogroups among which the nucleotide variation is more than 15% [17]. Traditionally, a highly variable region of 168 nucleotides within the viral genome encoding the VP1/P2A junction has been used for identifying and discriminating between different HAV strains [18]. However, several alternative regions within the genome including those encoding for VP1, 2C, and 3D also display high nucleotide variability and have offered limited alternative regions for strain identification [19]. Thus, whether tracking HAV strain(s) as they circulate through a given population or region, or linking a contaminated food item to an outbreak of illness, it is necessary and critical to accurately identify HAV strains by as few as one nucleotide variation along the entire viral genome. Whole genome sequencing (WGS) offers an approach by which single nucleotide differences/variations may be identified among strains. Inter-host HAV variants have been reported either under laboratory conditions or from clinical samples [20,21,22]. Vaughan et al. [23] completed the WGS analysis on HAV constructed from 16 PCR amplicons of 101 HAV strains as serum specimens from 4 food-borne outbreaks and 14 non-outbreaks. The whole genome data showed inter-host genetic diversity among the outbreaks and cases; however, analysis of intra-host HAV variants from eight patients of the same outbreak showed only a single sequence variant. In other words, intra-host variation was not commonly observed, which is likely due to the stringent negative selection preventing accumulation of SNVs during HAV infection [3].

Our previous study on HAV clone1-infected FRhK4 cells showed that both inter- and intra-host variants of only one single nucleotide difference existed in the absence of immune selection [24]. Although WGS offers the opportunity for accurate tracking of HAV strains and attributing the contamination sources, there have been no reports of intra-host variants from one infected single individual. This study extends our previous investigative approach, including pyrosequencing and next-generation sequencing (NGS), to the identification and discrimination of intra- and inter-host HAV variants from both cultured and clinical samples. We examined the relationship between the frequency of SNVs identified by NGS and the pyrosequencing confirmation by presenting a working model for HAV SNV calling from NGS data.

## 2. Materials and Methods

### 2.1. Viruses, Cell Culture, and Clinical Samples

The virus HAV HM175 clone 1 is a cell culture-adapted strain of wild-type HAV HM175 purchased from ATCC (American Type Culture Collection, VR-2089). Fetal rhesus monkey kidney FRhK-4 cell line persistently infected with clone 1 (defined as F4-c1 in this study) was established and maintained in our lab following the protocol previously described [25,26,27]. Persistently infected cells were sub-cultured in MEM-GlutaMax (GIBCO, Gaithersburg, MD, USA), supplemented with 1% pyruvate, 1% non-essential amino acids (GIBCO), and 5% heat-inactivated fetal bovine serum at 37 °C, and split at a ratio between 1:30 to 1:40, sufficient to yield a confluent culture weekly. The cells were periodically collected from 62 to 1200 days post-infection (dpi) and used for this investigation. The HAV HM175 positive human stool sample was obtained from Dr. Suzanne Emerson (National Institutes of Health).

### 2.2. Sample Preparation, RNA Isolation, and Viral RNA Quantification by RT-PCR (RT-qPCR)

F4-c1 cells were harvested at 100% confluency at different time points (62, 120, 180, 240, 335, 417, 500, 600, 800, 996, and 1200 dpi) as previously described [24,25]. In brief, cells were harvested from a T225 flask by scraping in culture medium, centrifuged at 1500× *g* for 30 min at 4 °C, cell pellets were washed and re-suspended in 2.5 mL cold phosphate-buffered saline (PBS) for subsequent use. Prior to RNA (termed as F4-C1 RNA in this study) isolation, cell pellets from each time point were lysed by being subjected to three rounds of freeze/thaw using a dry ice/methanol bath and a room temperature water bath, respectively. Complete disruption of cell integrity was determined by microscopic examination of a 1:1 dilution of the lysate in 0.4% trypan blue buffered solution (Gibco). Viral RNA was isolated from the F4-c1 cell lysates or HM175 stool supernatant in 10% PBS (vol/vol), respectively, with the QIAamp Viral RNA mini kit (Qiagen, Gaithersburg, MD, USA) following the manufacturer’s protocol. To determine the HAV genome copy numbers in the samples, one-step RT-qPCR was carried out following the protocol previously published [1,24]. In brief, all RNA samples were analyzed in replicates using QuantiTect Probe RT-PCR kit (Qiagen) with a 25 µL reaction volume containing 5 µL RNA. Ten-fold serial dilutions of an RNA transcript containing a complete HAV genome sequence generated from pHAV/7.1 as described previously by Yang et al. [24] were used to generate standard curves (RNA copy versus Ct). The reaction program included reverse transcription of RNA at 50 °C for 30 min, followed by a denaturation at 95 °C for 15 min, and finally 45 cycles of amplification (10 s at 95 °C, 25 s at 53 °C, and 25 s at 72 °C).

### 2.3. Library Generation and Sequencing

Double stranded cDNA libraries were generated from all the RNA samples above using a TruSeq stranded mRNA prep kit from Illumina (old Cat. No. RS-122-2101, new Cat. No. 20020594.) following our previously published protocol [1,24]. The total RNA input of each F4-C1 RNA sample ranged from 1–3 µg. The viral RNA input from the HM175 stool sample was 8.4 × 10^8^ copies. The libraries were validated for quality control by using the TapeStation (Agilent, Santa Clara, CA, USA), and for quantification by using Qubit (Thermo Fisher Scientific, Rockville, MD, USA). Barcoded libraries were pooled and sequenced on the MiSeq platform (Illumina, San Diego, CA, USA) with MiSeq Reagent kit (v2) to generate paired-end 100 base pair (bp) reads.

### 2.4. De Novo Assembly and Reference-Based Mapping

The raw read data in FASTQ files of all samples was imported from MiSeq into the CLC Genome Workbench v9.0 (CLC Bio, Aarhus, Denmark), and sequence quality was determined before further analysis. De novo assembly was performed to create a contig sequence from the raw reads to serve as a reference sequence. Reference-based mapping was carried out by mapping the raw reads against the specific reference sequence, as such, the read mapping could be used for the variant calling. For the samples in which the SNVs were investigated by both NGS and pyrosequencing, reference-based mapping was performed on the reads from the HM175 stool sample against the complete genome sequence of wild-type HAV HM175 (GenBank accession number M14707) and on the reads from 62 to 240 dpi samples against the HAV HM175 clone 1 sequence in NCBI (GenBank accession number M16632). Reads from the F4-c1 62 dpi sample (the earliest time point available in this study) were trimmed (quality score limit = 0.05, maximum number of ambiguities = 2) and de novo assembled to generate the viral genome using default parameters (Minimum contig length = 200 bps, minimum similarity = 0.8). The assembled contig, which was 7474 nt in length, was submitted as a BLAST query against the NCBI database and showed a 99% identity with the complete genome of HAV (attenuated) RNA (GenBank accession number M16632). For the F4-C1 RNA samples in which the SNVs were only investigated by NGS, this contig at 62 dpi was taken as the reference sequence; reference-based mapping was carried out on the reads of each sample from the time points afterward. In addition, the mapping parameters used had an 80% similarity over 50% of the read length as default. Total reads and mapped reads were summarized, and an estimated coverage was calculated to evaluate the overall coverage of the viral genome for each sample.

### 2.5. Single Nucleotide Variation Calling from NGS Reads and Confirmation by Pyrosequencing

Single nucleotide variation calling was carried out on each viral read mapping in CLC Genomics WorkBench. Since the estimated sequencing error rate with Illumina platform is approximated at 2% [28,29], to ensure high-quality and reliable variant calling, the parameters were set as a minimum coverage of 10 and minimum frequency of 2%. In addition, for some of the samples (62 to 240 dpi F1-C1 RNA, HM175 stool RNA), SNVs were also analyzed with pyrosequencing on an automated PSQ96MA instrument (Qiagen) following the protocol previously published [24,30,31]. First, reverse transcription was carried out on the viral RNA samples using oligo(dT) primer and AMV reverse transcriptase that is able to amplify up to 7.8 kb or longer cDNA according to the company (Promega, Madison, WI, USA). Second, PCR with flanking primers (Supplementary Table S1a) was performed to amplify the amplicons ranging from 148 to 553 bp in length to cover the genomic regions of interest. Third, the PCR templates were biotinylated (20 µL) and immobilized onto 3 µL streptavidin-coated Sepharose beads (GE Health care Biosciences, Uppsala, Sweden) in a 96-well plate, then, the bead-PCR product was transferred onto a filter with a vacuum prep tool followed by washes for 5 s each with 70% ethanol, 0.2 M NaOH, and washing buffer 10 mM Tris-acetate, pH 7.6, respectively. The beads were then released and resuspended in an annealing buffer (40 µL) containing 4 µL of the respective sequencing primers (Supplementary Table S1b) in a 96-well plate. Pyrosequencing was performed in SNP (single nucleotide polymorphism) modes using the PSQ reagent kit according to the manufacturer’s instructions to generate short sequences (10–15 nucleotides). Specific nucleotides were added to the sequencing-primer-bound ssDNA (single-stranded DNA), and the light signal associated with the identity of the incorporated nucleotides was recorded.

### 2.6. GenBank Accession Number

The data from Illumina sequencing have been deposited in the NCBI Sequence Read Archive (www.ncbi.nlm.nih.gov/sra) under accession number SRP118687 (BioProject PRJNA408289).

## 3. Results

Our previous study revealed that an intra-host heterogeneity existed in virus-infected cultured cells detected by NGS and was confirmed with pyrosequencing [24]. Pyrosequencing methods are well-established and have proven suitable for SNV analysis and viral SNV identification [32,33]; this technology is capable of detecting and quantifying allelic frequency to as low as ~5% [34]. We also concluded in our previous study that both read coverage and nucleotide frequency at a given position are significant for the SNV calling from NGS data and pyrosequencing confirmation [24]. The requirement of sequencing coverage varies and depends on the specific applications. For example, in determining the sequence variation of SNVs and small indels (insertions and deletions), it usually requires an average depth of 15× and 33× to detect homozygous SNVs and the same proportion of heterozygous SNVs, respectively, based on the studies on human genome sequencing with the Illumina platform [35,36]. Consequently, an average coverage depth of 50× was suggested for the requirement to allow reliable SNV calling along 95% of the genome [37]. In the current investigation, each of the SNVs selected for confirmation by pyrosequencing had a relatively high depth of nucleotide coverage (≥93×) and high base quality (Phred quality score > 30, or the base call accuracy > 99.9%), thus, the results in this study showed mainly the relationship between SNV frequency from NGS and the SNV validation with pyrosequencing.

We first sought to determine whether intra-host variation(s) exist in HAV from clinical samples. Illumina MiSeq reads from a HAV HM175 positive stool sample were mapped against the wild-type HM175 reference sequence (GenBank accession number M14707); eight SNVs were detected with frequencies ranging from 13.8–99.7%. The depth of the read coverage at each nucleotide position was ≥24,000× (Table 1). These SNVs were identified by the NGS variant caller as either “single-SNV” or “mixed-SNVs”. In the case of single-stranded genomes such as HAV, “single-SNV” are defined as having only one major variant called at that position, and “mixed-SNVs” are defined as having more than one variant called at that position. To validate the SNVs called from NGS, pyrosequencing was carried out on all eight SNVs. The SNVs at positions 2864, 4185, 5204, 6216, and 6522 had variant frequencies ranging from 81.1–99.7 and were all identified as single-SNV (i.e., inter-host variations) by pyrosequencing (Table 1). In contrast, two of these five SNVs (at positions 5204 and 6216) were called by NGS as “mixed-SNVs”. On the other hand, SNVs at positions 1742 and 6836 with frequencies of 54.2 and 40.1 were both called as mixed-SNVs and, therefore, represent intra-host variations (Supplementary Figure S1). The insertion at 7042 with a frequency of 13.8 was not called as a real SNV by pyrosequencing.

**Figure 1:**
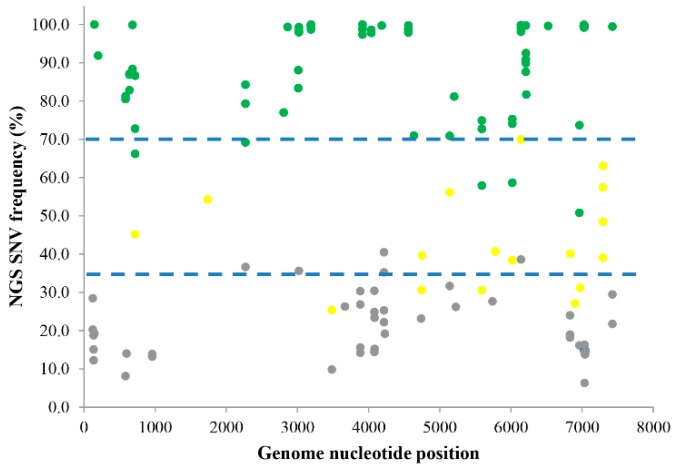
Comparison of SNV calling between NGS and pyrosequencing. Each dot represents an individual SNV identified by NGS in extracts from either F4-c1 culture (62 to 240 dpi) against the reference sequences M16632, or the HAV HM175 positive clinical sample against the reference sequence M14707. Phred quality score >30 for each SNV (*p* < 0.001 for the error rate). SNVs were validated by pyrosequencing as a single-SNV (green), mixed-SNVs (yellow), or a non-SNV (grey). The dotted blue lines were artificially drawn to delineate different groups containing the majority of single-, mixed-, and non-SNVs, respectively. The *X*-axis represents the nucleotide position along the respective reference genomes. The *Y*-axis represents the frequency of each SNV from NGS results.

To investigate the relationship between the frequency of SNV calling from NGS and the confirmation of SNVs with pyrosequencing, first, NGS was performed and SNVs were called on the clinical sample (against reference M14707) and cultured F4-c1 samples at time points of 62 to 240 dpi (against reference M16632). One hundred and thirty-four SNVs with the average coverage ranged from 93–5324× were detected by NGS (Figure 1). Pyrosequencing was then performed on each of these SNVs. Seventy-one SNVs were identified by pyrosequencing as single-SNVs, and 66 (93.0%) of them had NGS frequencies ranging from 70.9 to 100%; 17 SNVs were identified as mixed-SNVs, and 12 (70.6%) of them had frequencies ranging from 35.2 to 69.9%; 46 SNVs were not validated by pyrosequencing, and 41 (89.1%) of them had frequencies ranging from 6.3 to 33.5%. Thus, based on our current data and graphical analysis (Figure 1), an SNV from NGS with a frequency >70% could be validated by pyrosequencing as a single-SNV with a 93.0% probability; an SNV from NGS with a frequency between 35–70% could be validated by pyrosequencing as a mixed-SNVs with a 70.6% probability; however, if an SNV called by NGS has a frequency <35%, then there is an 89.1% possibility it would not be validated as a real SNV by pyrosequencing. We used this relationship as a model to predict the single-SNV, mixed-SNVs, and non-SNVs in the following experiments based on their NGS frequencies.

**Figure 2:**
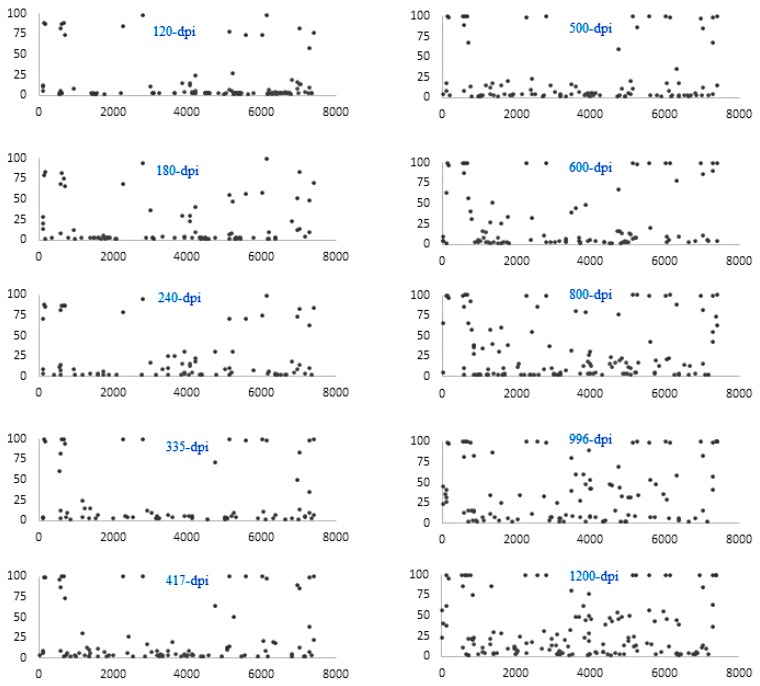
Distribution of single nucleotide variants across the F4-C1 genome at different time points. Each dot represents each SNV identified by NGS at the nucleotide position along the mapped reference sequence of F4-C1 genome at 62 dpi (*x*-axis) with its variant frequency (*y*-axis) at that position. Only those variants with a frequency >2% are represented in the graphs.

**Figure 3:**
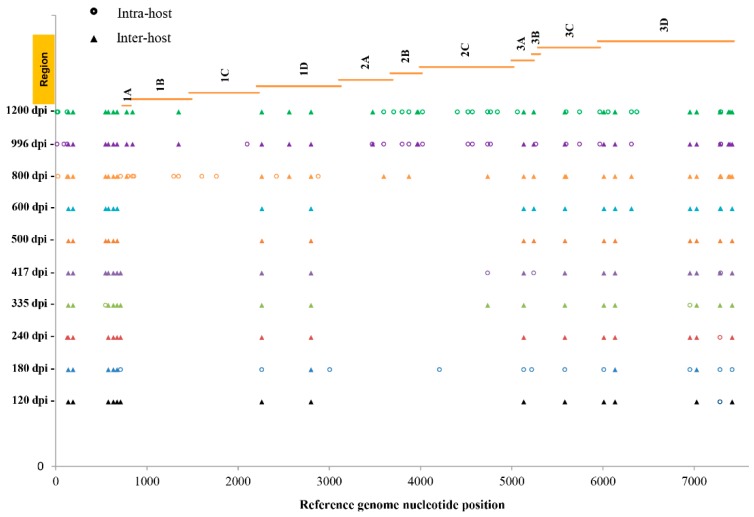
SNVs in F4-C1 samples over time post-infection identified by NGS and predicted as intra- or inter-host variants based on the NGS frequency model. SNVs were detected from each time point 120 to 1200 dpi (represented at different colors) against 62 dpi F4-C1 RNA sample, and variant frequencies determined and assignment as intra-, inter-, or non-SNV variants as listed in Supplemental Table S2. Each triangle represents one SNV called by NGS and predicted as a single-SNV, and each circle represents one SNV called by NGS and predicted as mixed-SNVs. The *X*-axis represents the nucleotide position along the mapped reference sequence of F4-C1 genome at 62 dpi. Orange lines in the top panel represent various regions along the HAV genome: 5’-UTR (1–734 nt), capsid proteins 1A to 1D (735–3107 nt), non-structural proteins 2A to 3D (3708–7415 nt). 2C: ATPase (predicted); 3B: VPg; 3C: Protease; 3D: RNA-dependent RNA polymerase.

**Figure 4:**
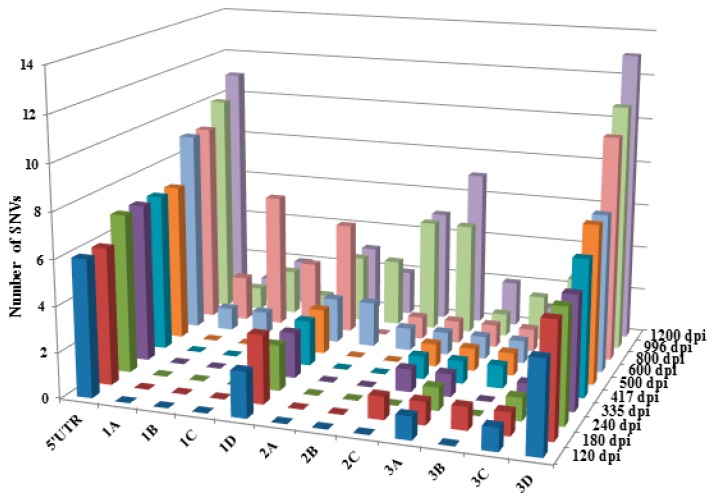
Distribution of SNVs along the HAV genome in F4-c1 samples. HAV SNVs in F4-c1 samples were detected from each time point 120 to 1200 dpi (represented as different colors) against 62 dpi. *X*-axis represents different regions (not in proportion) along the mapped reference sequence of F4-C1 genome at 62 dpi. *Y*-axis represents the number of SNVs in each region. *Z*-axis represents the different time points at days post infection.

To investigate the occurrence, distribution, and persistency of SNVs over time in the F4-c1 sample extracts, SNVs from 120 to 1200 dpi were compared to those seen in the samples from the earliest time point available (62 dpi). For each sample, there were 72–146 SNVs called by NGS with a frequency >2% (Figure 2). Since the possibility for a NGS SNV with a frequency less than 35% to be confirmed as a non-SNV by pyrosequencing was 89.1% based on our current model, and to make the list more concise and clear, only the SNVs with frequencies >10% (a total of 442 SNVs) were included in the analysis (Supplementary Table S2). Each SNV had a Phred quality score >30, and the average coverage ranged from 51–2558×, except for the SNVs (single-SNV) at 7393 and 7417 nt positions in 996 and 1200 dpi samples. These SNVs had a coverage of 42, 22, 47, and 34×, respectively, but were still higher than the requirement of an average depth of 15× for detection of “homozygous” SNV (single-SNV) [35,36]. All the SNVs were further predicted as either (182 out of 442 = 41%) single-, (93 out of 442 = 21%) mixed-, or (167 out of 442 = 38%) non-SNVs based on their NGS frequencies (Supplementary Table S2). As summarized in Figure 3 (predicted non-SNVs were not included), (i) SNVs were detected across the whole genome, including regions of UTRs (untranslated regions), structural proteins, and non-structural proteins; (ii) an SNV detected at a nucleotide position from an early time point could be persistently present in the samples from later time points; (iii) both mixed-SNVs (intra-host) and single-SNV (inter-host) at a position were detected in samples from each time point; (iv) a majority of the mixed (intra-host) SNVs from earlier time points would become single-SNV (inter-host) in later time points, with a few of exceptions, such as the ones at nucleotide positions of 3005 and 4209; (v) the number of SNVs increased in samples starting from 600 dpi, which was also shown in Figure 4; (vi) SNVs from 62 nucleotide positions along the genome were detected and identified, based on the model in the current study. Further analysis found that 12 out of these 62 SNVs were noncoding SNVs, and the remaining 50 SNVs fell inside the protein-coding region (Supplementary Table S2). Among these 50 SNVs in the coding region, 39 of them were synonymous, and only 11 of them were nonsynonymous, which resulted in a change in the amino acid.

## 4. Discussion

For virologists working with foodborne viruses, achieving strain identification/ discrimination at the level of single nucleotide differences/point mutations with a concomitant level of confidence assigned to those differences remains an important goal. This level of discrimination would greatly aid in not only strain identification, but also in outbreak investigation, regulatory surveillance, and perhaps source attribution. Indeed, tracking virus strains by as few as one nucleotide variation could be accomplished by whole genome sequencing. However, accurate identification of SNVs is challenged by errors generated during NGS. Error reduction could be achieved by various strategies, such as increasing the depth of reads coverage [37], or developing and improving bioinformatic tools for data analysis and error identification, particularly for nucleotide variation [29,38,39]. In this study, in addition to ensuring the high depth of coverage for the SNVs, we employed both pyrosequencing and Illumina NGS for the SNV identification to combine both sequencing methods with complementary strengths. Pyrosequencing is a real-time sequencing method which is based on the detection of pyrophosphate released after each nucleotide incorporation in the new synthetic DNA strand, optimal for sequencing and analysis of short stretches of DNA, including SNPs [30], and has been intensively used for SNP detection since its emergence in 2005 [32,33,34]. Illumina NGS is one of the more recent sequencing technologies, it is massively parallel and allows millions of fragments to be sequenced in a single run, and it is currently used for wide applications including variation detection.

The results showed (Supplementary Table S2) that some SNVs were repeatedly detected in a series of F4-C1 samples by NGS, but not detected by pyrosequencing. For example, an SNV at nucleotide position 1185 was called at 335 to 600 dpi with the frequency <29.7%, then disappeared at later time points. The explanations for this observation could be: (1) it was a random and/or repetitive NGS error in SNV identification rather than a real variation, or (2) it was a real SNV at early time points, but the mutation rate was lower than the pyrosequencing detection sensitivity (~5% allele frequency), and thus it could not be confirmed. Additional explanation for the absence of this SNV at later time points might be a result of negative selection. The latter interpretation is supported by another observation in our study. For example, the SNV at nucleotide position 1348 was repeatedly called from 417 to 1200 dpi by NGS with various frequencies. According to our NGS frequency model, pyrosequencing would confirm it as a non-SNV at 417 and 500 dpi (NGS frequencies of 10.8% and 17.6%, respectively), a mixed-SNVs at 600 and 800 dpi (NGS frequencies of 51.1% and 40.7%, respectively), and a single-SNV at 996 and 1200 dpi (NGS frequencies of 87.1% and 86.6%, respectively). The possibility would be a real variation at this position started from 417-dpi with a very low mutation frequency, then became the major and eventually the dominant type due to the positive selection. Similarly, the SNVs called by NGS with lower frequencies from earlier time points also occurred at other positions. These single nucleotide mutations either were eliminated or became single-SNVs at later time points, likely due to different selective pressures on the replication and maintenance of the viral genome population. In addition, our conclusions are also consistent with the findings in a study on hepatitis C virus (HCV) infection from early stage to resolution of disease outcome [40], indicating the existence of rare variants at frequencies at or below the detection threshold. In response to the selection pressure, the frequencies of these rare variants can increase rapidly.

It should be noted that only 12 out of 17 (70.6%) SNVs with NGS frequencies ranging between 35 to 70% were confirmed as mixed-SNVs by pyrosequencing and included in the current study. Thus, for this specific model, a mixed-SNVs type of SNV was predicted with only a 70.6% probability to be validated by pyrosequencing. Taking the small sample size (*n* = 17) into account, we believe that when a new model is re-created, the SNV detection accuracy and the power of the model based on the NGS frequencies could increase by increasing the sample number (the cutting-off frequency % between groups could be altered accordingly), especially for the mixed-SNVs group.

It has been commonly believed that the 5′- and 3′-UTRs of HAV are highly conserved regions [41,42], and the coding region usually exhibits a high genetic diversity [43,44]. Indeed, our results obtained from the stool sample extract demonstrated that all seven confirmed SNVs fell within the polyprotein coding region and with only one SNV (at position 2864 nt) located in the VP1-P2A junction region. These results are consistent with the previous finding that, besides the VP1-P2A junction, other regions across the genome also display nucleotide variability [19]. Interestingly, our results of the cultured F4-c1 samples indicated that SNVs are distributed along the whole genome, in both UTRs and coding regions. In fact, the more extensive nucleotide variants were observed in both 5′-end (5′-UTR) and 3’-end (3D regions, RNA polymerase), compared with other individual coding regions (Figure 4). Previous studies demonstrated that the 5’ proximal regions of the uncapped genome of picornaviruses have an internal ribosome entry site (IRES) and are involved in translation as well as RNA synthesis [45,46]. In addition, a stem-loop structure predicted in the 5’ proximal region of red clover necrotic mosaic virus played important host-dependent roles in both translation and RNA stability [45]. Thus, the diverse structure of the 5′ proximal region of the positive-sense single-stranded RNA viruses is one of the strategies of the viruses to exploit host resources to perform their own preferential translation or proper translation regulation [47,48]. In the current study, the pattern of SNV distribution between the wild-type stool sample and cultured F4-c1 samples differed from each other. More genetic variations in HAV 5’-UTR, in combination with the 3D region variations (RNA polymerase), might play roles for the virus translation, rapid proliferation, and culture adaptation in FRhK4 cells.

The original clone 1 strain that infected the FRhK4 cells, and samples from earlier time points prior to 62 dpi were unavailable for inclusion in this investigation. Therefore, HAV evolution, amino acid function change, and mutation rate are beyond the extent of this study.

In summary, our study identified inter- and intra-host variants of HAV in different environments and demonstrated the co-existence of inter- and intra-host variants both in the clinical specimen and under laboratory culture conditions. Our findings of intra-host variants from the clinical sample demonstrated the presence of multi-viral RNAs in a single infected individual. This is significantly important for the discrimination of strains at the SNV level, outbreak investigations, and source attribution. In other words, if more than one HAV molecule with only a few nucleotide differences are detected from one food item, it could be from multiple contamination sources, but the possibility of a single contamination source should not be excluded, and thereby further investigation is needed. Additionally, the detection of HAV genetic variability using NGS, which is likely more sensitive than traditional pyrosequencing, has improved our understanding of the basis of control on intra- and inter-host population dynamics. As such, the fate of mutations could be determined by different selective pressures. A minority mutation, starting and maintaining at a low frequency, may either be eliminated as the result of negative selection, or become a major type through positive selection. Our study also suggested that whole-genome sequencing could potentially provide a valuable approach to the studies, not only in HAV but also in other viruses, for the accurate identification and source attribution at SNV level.

## Figures and Tables

**Table 1:** Identification of single nucleotide variations (SNVs) present in viral RNA extracted from a Hepatitis A virus (HAV) HM175 positive stool sample. NGS = next-generation sequencing.

Reference Position ^a^	Coding Region	Reference ^b^	Change ^c^	Coverage ^d^	Frequency ^e^	SNV Called by NGS ^f^	Amino Acid Change ^g^	SNV Identified by PYROSEQUENCING
1742	1C	G	A	22,779	54.2	mixed		mixed
2864	1D	T	A	24,773	99.3	single		single
4185	2C	G	A	69,820	99.7	single	AAA45465.1:p.[Glu1151Lys]	single
5204	3A	G	A	138,614	81.1	mixed		single
6216	3D	T	C	166,496	81.7	mixed		single
6522	3D	T	A	297,276	99.6	single	AAA45465.1:p.[Ser1930Thr]	single
6836	3D	C	T	210,792	40.1	mixed		mixed
7042	3D	-	A	260,563	13.8	mixed	AAA45465.1:p.[Gln2103fs]	

^a^ Nucleotide position in reference sequence M14707; ^b^ The reference sequence at the position of the variant; ^c^ The changed sequence of the variant; ^d^ The depth of NGS coverage at that nucleotide position; ^e^ The read count of variant at that nucleotide position divided by coverage; ^f^ The SNVs called at this position by the variant caller. Single-SNV: only one variant called at that position; mixed-SNV: more than one variant called at that position; ^g^ Amino acid change in the coding region of the hepatitis A virus polyprotein AAA45465.1.

## Supplementary Materials

The following are available online at http://www.mdpi.com/1999-4915/10/11/619/s1, Inter- and intra-host nucleotide variations of hepatitis A virus in culture and clinical samples detected by next-generation sequencing. Figure S1: the representative pyrogram of the heterozygosities. Table S1 (a) initial PCR primers for amplicons, (b) sequencing primers for pyrosequencing. Table S2: summary of F4-C1 variants called by NGS from multiple time points against 62-dpi.
