# Supplementary material for: Inter- and Intra-Host Nucleotide Variations in Hepatitis A Virus in Culture and Clinical Samples Detected by Next-Generation Sequencing
Source: Viruses. 2018 Nov 9;10(11):619. doi: 10.3390/v10110619 (PMC6265925; doi:10.3390/v10110619)
Supplement: Supplementary file 1 [file viruses-10-00619-s001.zip › Supplementary figure 1.pptx]

## Slide 1
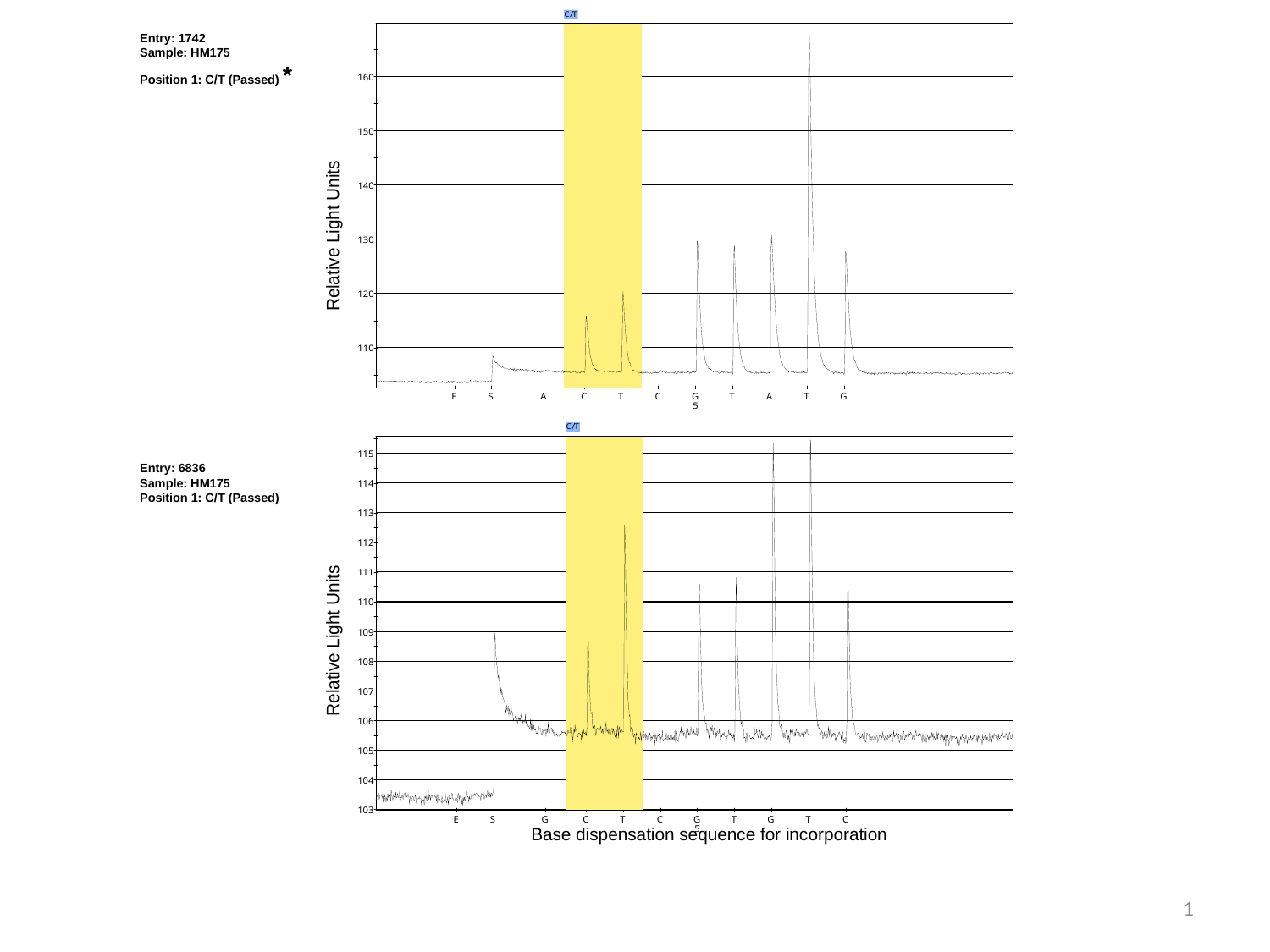

Entry: 1742
Sample: HM175
Position 1: C/T (Passed) *
Relative Light Units
Entry: 6836
Sample: HM175
Position 1: C/T (Passed)
Relative Light Units
Base dispensation sequence for incorporation
1

## Slide 2
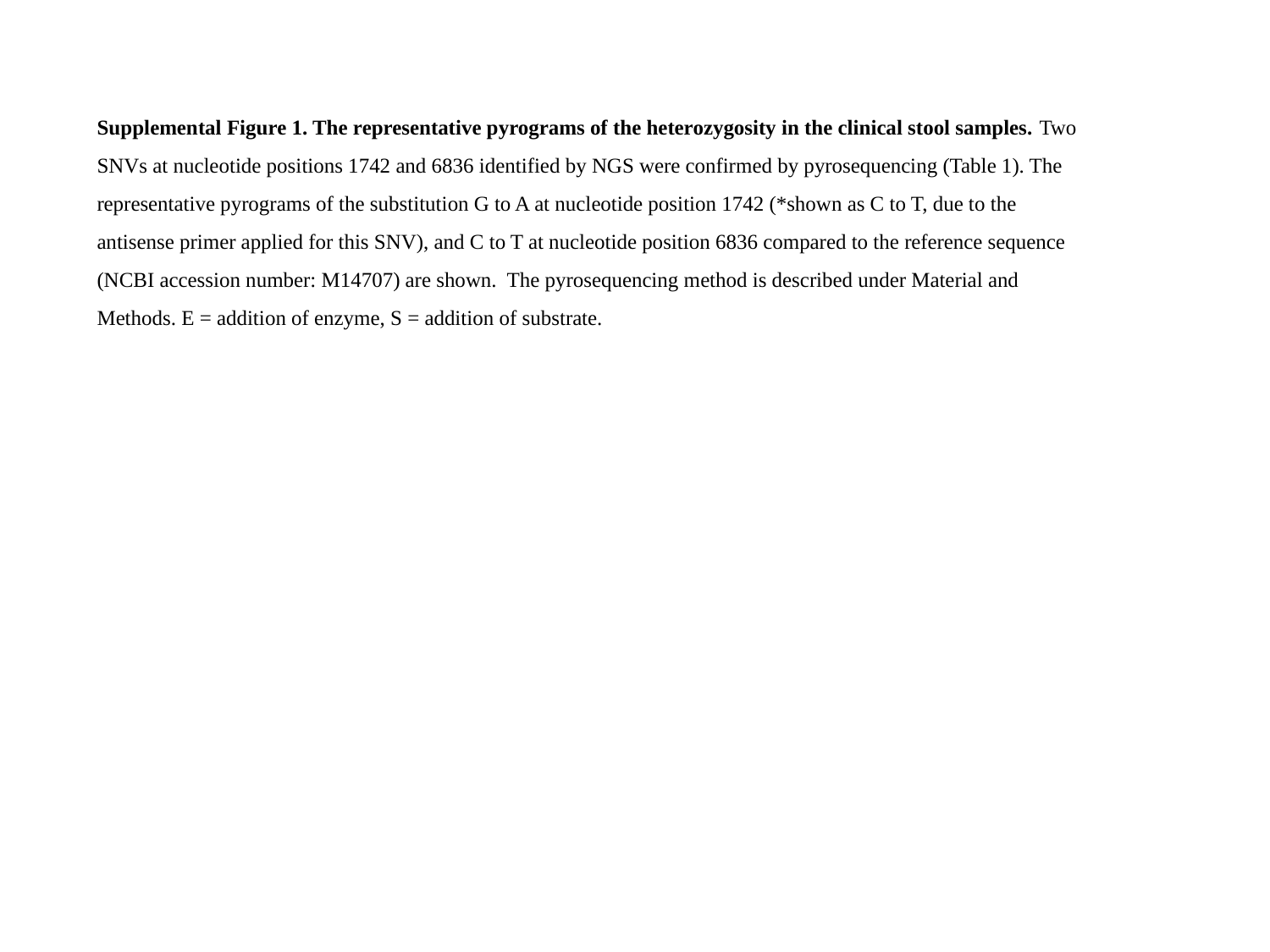

Supplemental Figure 1. The representative pyrograms of the heterozygosity in the clinical stool samples. Two SNVs at nucleotide positions 1742 and 6836 identified by NGS were confirmed by pyrosequencing (Table 1). The representative pyrograms of the substitution G to A at nucleotide position 1742 (*shown as C to T, due to the antisense primer applied for this SNV), and C to T at nucleotide position 6836 compared to the reference sequence (NCBI accession number: M14707) are shown. The pyrosequencing method is described under Material and Methods. E = addition of enzyme, S = addition of substrate.
